# Clinical relevance of RNA editing profiles in lung adenocarcinoma

**DOI:** 10.3389/fgene.2023.1084869

**Published:** 2023-03-10

**Authors:** Si Shi, Shibin Chen, Menghang Wang, Bingchen Guo, Yaowu He, Hong Chen

**Affiliations:** ^1^ The Respiratory Department, The Second Affiliated Hospital of Harbin Medical University, Harbin, Heilongjiang, China; ^2^ Medical Research Center, Beijing Chao-Yang Hospital, Capital Medical University, Beijing, China; ^3^ Department of Cardiology, The first Affiliated Hospital of Harbin Medical University, Harbin, Heilongjiang, China

**Keywords:** lung adenocarcinoma, RNA editing, overall survival, nomogram, prognostic model

## Abstract

**Background:** Lung adenocarcinoma (LUAD) is the most frequently occurring lung cancer worldwide, with increasing death rates. It belongs to the non-small cell lung cancer (NSCLC) type and has a strong association with previous smoking history. Growing evidence has demonstrated the significance of adenosine-to-inosine RNA editing (ATIRE) dysregulation in cancer. The aim of the present study was to evaluate ATIRE events that might be clinically useful or tumorigenic.

**Methods:** To explore survival-related ATIRE events in LUAD, its ATIRE profiles, gene expression data, and corresponding patients’ clinical information were downloaded from the Cancer Genome Atlas (TCGA) and the synapse database. We evaluated 10441 ATIRE in 440 LUAD patients from the TCGA database. ATIRE profiles were merged with TCGA survival data. We selected prognostic ATIRE sites, using a univariate Cox analysis (*p* < 0.001). Cox proportional hazards regression and lasso regression analysis were used to determine survival-related ATIRE sites, create risk ratings for those sites, and build a prognostic model and a nomogram for assessing overall survival (OS). Six ATIRE sites were used in the prognostic model construction and patients were randomly divided into a validation cohort (*n* = 176) and a training cohort (*n* = 264). The “Pheatmap” program was used to create risk curves that included risk score, survival time, and expression of ATIRE sites. We also determined the clinical prediction model’s discrimination. The decision curve analysis and the 1-, 2-, and 3-year corrective curves were simultaneously used to evaluate the nomogram. We also evaluated the relationship between the amount of ATIRE sites and host gene expression and the impact of ATIRE expression on transcriptome expression.

**Results:** The pyroglutamyl-peptidase I (PGPEP1) chr19:18476416A > I, ankyrin repeat domain 36B pseudogene 1 (ANKRD36BP1) (dist = 3,795), T-box transcription factor (TBX19) (dist = 29815) chr1:168220463A > I, Syntrophin Beta 2 (SNTB2) chr16:69338598A > I, hook microtubule-tethering protein 3 (HOOK3) chr8:42883441A > I, NADH dehydrogenase flavoprotein 3 (NDUFV3) chr21:44329452A > I, and FK506-binding protein 11 (FKBP11) chr12:49316769A > I were used in the prognostic model construction. High levels of risk score were significantly associated with worse OS and progression-free survival. Tumour stage and risk score were related to OS in LUAD patients. The predictors were among the prognostic nomogram model’s risk score, age, gender, and tumor stage. The calibration plot and C-index (0.718) demonstrated the significant accuracy of nomogram’s predictions. ATIRE level was markedly elevated in tumor tissues and was highly variable between patients.

**Conclusion:** Events involving ATIRE in LUAD were highly functional and clinically relevant. The RNA editing-based model provides a solid framework for further investigation of the functions of RNA editing in non-coding areas and may be used as a unique method for predicting LUAD survival.

## 1 Introduction

Lung cancer has become one of the most common cancers worldwide, particularly the primary histological subtype adenocarcinoma (LUAD). It rapidly develops and has a poor clinical outcome. Although numerous targeted drugs have been used to treat lung adenocarcinoma, their efficacy is still not satisfactory. Therefore, it is important to describe predictive molecular markers and pinpoint the molecular changes that underlie LUAD. Epigenetic modifications play an important role in the development of lung cancer and represent one of the main mechanisms for the acquisition of resistance to targeted therapy in non-small cell lung cancer (NSCLC). Typical epigenetic controls include DNA methylation, histone modification, non-coding RNA control, and chromatin remodeling, which control gene expression and maintain genome stability without altering DNA sequence (Nebbioso et al., 2018). As an epigenetic mechanism, RNA editing is closely related to the pathogenesis of various cancers.

RNA editing is a molecular technique to alter RNA. Insertion, deletion, or base substitution of nucleotides modifies the primary RNA transcripts (Su and Randau, 2011). RNA editing is a posttranscriptional mechanism conserved in metazoans, which comprises A to I deamination in RNA by adenosine deaminases that act on RNA (ADARs) (Huntley et al., 2016) and cytosine (C) to uracil (U) deamination by the apolipoprotein B mRNA editing enzymes, catalytic polypeptide-like (APOBEC) enzymes (Sharma et al., 2015). APOBEC is a potent restriction factor that represses retroelements after reverse transcription through cytosine-uridine editing of retroelement DNA (Sharma et al., 2015). With millions of editing sites already identified in humans, A-to-I RNA editing, mediated by ADAR family of enzymes, is regarded as the most prevalent RNA alteration in mammals (Bazak et al., 2014a). Most cancer types frequently show increased A-to-I RNA editing and the enzymes underlying this transformation. Chigaev et al., showed that RNA editing levels were elevated in malignancies. A-to-I RNA editing has significant functional effects across the whole genome, particularly as a cancer-related non-coding RNA regulator (Chigaev et al., 2019). Protein-coding RNA and non-coding RNA are subject to RNA editing. According to Paz-Yaacov et al., enzymatic changes in RNA sequences may also have a role in cancer etiology (Paz-Yaacov et al., 2015). A-to-I RNA editing contributes to proteomic diversity in breast cancer through changes in amino acid sequences (Peng et al., 2018) and are increased in most tumor tissues examined, which may be associated with ADAR1 overexpression (Xu et al., 2018; Fritzell et al., 2019). A high frequency of site-specific RNA editing events, substitution of acylglycine by serine at residue 367 (S367G) in antienzyme inhibitor 1 (AZIN1) was found in hepatocytes, LUAD, and esophageal carcinoma (Chen et al., 2013; Qin et al., 2014; Hu et al., 2017). Wang et al. systematically characterized the miRNA editing profiles of 8,595 samples from 20 cancers derived from The Cancer Genome Atlas (TCGA) miRNA sequencing data and correlated patient outcomes with miR-200b editing levels. They demonstrated the importance of miRNA editing in gene regulation and its importance in cancer detection and treatment (Wang et al., 2017a). In addition, Paz-Yaacov et al. showed that most cancer types significantly modify RNA editing and ADAR expression and that increased editing activity was related to patient survival (Paz-Yaacov et al., 2015). Taken together, some RNA editing events may serve as prognostic or predictive indicators for patient stratification and “drivers” for the development of tumors.

Targeted therapeutics for RNA editing will be required as the role of RNA editing in human disease is clarified (Kung et al., 2018). To forecast the overall survival (OS) and progression-free survival (PFS) of people with LUAD, we created a prediction model. By examining all RNA editing profiles and clinical information for LUAD in the TCGA database, we identified RNA editing events associated with OS. We then created a nomogram to predict the prognosis of LUAD based on the RNA editing risk score. We also examined these ATIRE sites underlying processes that affect LUAD survival.

## 2 Materials and methods

### 2.1 Data collection

We evaluated 54 normal and 504 LUAD samples from the TCGA database (TCGA, https://portal.gdc.cancer.gov/), along with the RNA sequence and associated clinical data. We examined the ATIRE profiles of TCGA-LUAD samples, which were downloaded from the synapse website (https://www.synapse.org/#!Synapse:syn2374375/files/). Sites that had less than 5% editing in over 90% of samples and regular samples were eliminated. The 440 samples included in the study were randomly split into a training cohort (*n* = 264) and a validation cohort (*n* = 176), based on the amount of available ATIRE data for each sample.

### 2.2 Construction of a prognostic model

Considering all patients as a combination cohort (*n* = 440), they were further separated into the validation (*n* = 176) and the training (*n* = 264) cohorts at a ratio of 5–3. Data from the training cohort were verified in the validation and the combination cohorts prior to the construction of the prediction model. ATIRE sites significantly (*p* < 0.001) correlated with the OS, and they were initially screened through the univariate Cox regression analysis on the training cohort. We applied the Least Absolute Shrinkage and Selection Operator (LASSO) regression model in the study of the multivariate Cox proportional hazard regression. By applying univariate and multivariate Cox regression, we found six distinct ATIRE sites that were linked with prognosis, whose coefficients and expression levels were then used to construct the risk score model. The risk score for each LUAD patient was calculated by the following formula: 1*Exp1 + 2*Exp2 + i*Expi, where the Exp preceding number is the coefficient value of the independent prognosis-associated ATIRE sites, Exp is their expression level, and “i” is the number of ATIRE sites.

### 2.3 Assessing the RNA editing profiles prognostic risk model

We used the median risk score to classify LUAD patients into low- and high-risk groups for the training and validation cohorts. The “survminer” and “survival” packages in the R software were used to conduct the survival analysis of ATIRE sites expression and risk values. We conducted ATIRE sites expression, survival time, and risk curves including the risk score, using the pheatmap’ package. Applying the ‘survival’ package and the Pearl software, an independent prognostic analysis was performed according to the clinical information and the risk scores of each sample. Through the multivariate and univariate Cox analyses, we determined risk scores, tumor (T), node (N) states, metastasis (M), gender, and age. ‘Survival ROC’ package from the R software was used to analyze the receiver operating characteristic (ROC) curve.

### 2.4 Validating the performance of the prognostic model

Patients lacking complete clinical information, including tumor, node, and metastasis (TNM) stage, gender, and age, were excluded as we determined the predictive effect on the model constructed. Furthermore, we used the R packages ‘Forestplot’ and ‘Survival’ to include clinical indicators and risk scores into multivariate and univariate Cox regression analyses. Based on these, the 1-, 2-, and 3-year survival of LUAD patients were predicted by a nomogram constructed with the ‘Rms’ package. To assess the performance of the nomogram, we determined the decision curve analysis (DCA), the calibration, and the time-dependent ROC analyses. In addition, we evaluated the concordance indices (C-index) using R.

### 2.5 Gene ontology (GO) enrichment and kyoto encyclopaedia of genes and genomes (KEGG) pathway analyses

We further investigated the bioinformatics of aberrantly expressed RNA editing and differences in molecular mechanisms and functional pathways between high-risk and low-risk patients. The expression status of certain genes in specific functional gene sets was examined using Gene set enrichment analysis (GSEA). The “c2. cp.kegg.v2022.1. Hs.symbols.gmt” and “c5. go.v2022.1. Hs.symbols.gmt” KEGG gene sets were obtained from the Molecular Signatures Database were used to detect pathways and molecular mechanisms. We applied GSEA to differentiate the biological functions of both groups and conducted the KEGG pathway and GO enrichment analyses, using the ggplot2, enrichplot, clusterProfiler, and DOSE from the R packages, and others, to systematically examine the biological functions of the differentially expressed genes between high-risk and low-risk groups. False discovery rate (FDR) and *p* values <0.05 were statistically significant.

## 3 Results

### 3.1 Screening prognostic-related ATIRE site and constructing a prognostic risk model

**FIGURE 1:**
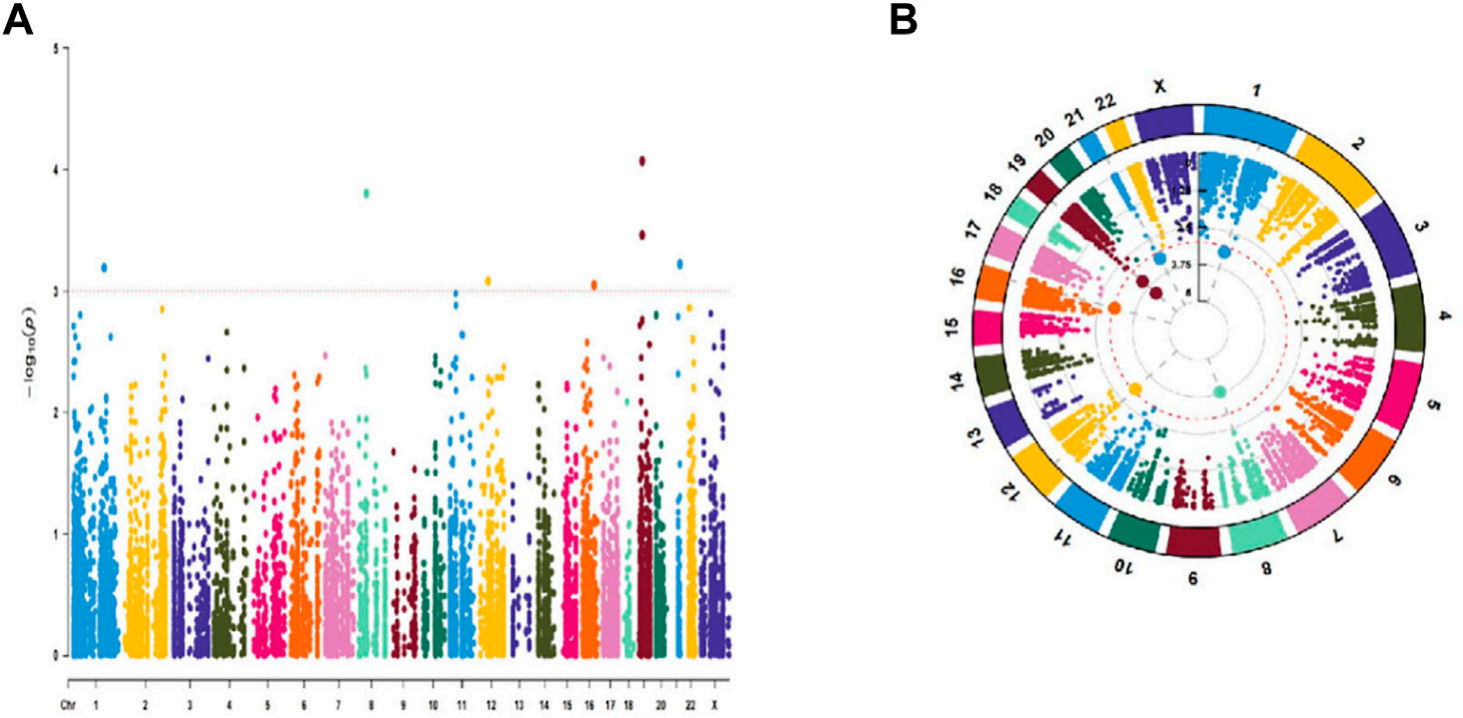
Univariate Cox regression analysis of 10441 ATIRE sites that were significantly correlated with OS in the training cohort. **(A)** Manhattan diagram of 10441 ATIRE sites. **(B)**Circle graph of 10441 RNA editing sites.

**FIGURE 2:**
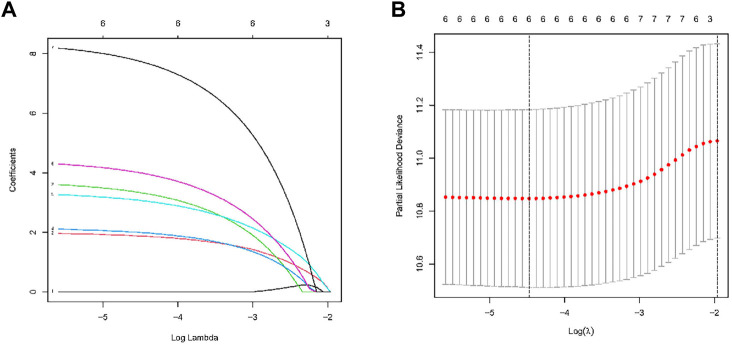
RNA editing sites selection using the LASSO model and multivariable Cox model.**(A)** Ten-fold cross-validation for the coefficients of six ATIRE sites in the LASSO model. **(B)** A coefficient profile plot was produced against the log (lambda) sequence in the LASSO model. The optimal parameter (lambda) was selected as the first black dotted line indicated.

The TCGA comprised 440 LUAD patients with complete OS data for the follow-up research. The complete dataset was separated into validation (*n* = 176) and training (*n* = 264) cohorts, as we were defining the entire dataset as the combination cohort. We evaluated 10441 ATIRE sites in the 440 LUAD patients from the TCGA database. Seven ATIRE sites with OS by conducting the univariate Cox regression analysis on the ATIRE sites expression profiles in the training cohort significantly (*p* < 0.001) correlated and 10441 ATIRE sites were visualized using circle plots by Manhattan diagram and circle graph (Figures 1A, B). By estimating the prediction accuracy of about 1000 cross validations, we performed the LASSO regression on these ATIRE sites for preventing the over-fitting of the model (Figures 2A, B).

We used the ATIRE sites the pyroglutamyl-peptidase I (PGPEP1) chr19:18476416A > I, ankyrin repeat domain 36B pseudogene 1 (ANKRD36BP1) (dist = 3,795), T-box transcription factor (TBX19) (dist = 29815) chr1:168220463A > I, Syntrophin Beta 2 (SNTB2) chr16:69338598A > I, hook microtubule-tethering protein 3 (HOOK3) chr8:42883441A > I, NADH dehydrogenase flavoprotein 3 (NDUFV3) chr21:44329452A > I, and FK506-binding protein 11 (FKBP11) chr12:49316769A > I in the prognostic model construction, as well as the corresponding coefficients (Table 1). The final risk score calculation formula was as follows: Risk score = expression value of PGPEP1|chr19:18476416 * (2.005077) + expression value of ANKRD36BP1 (dist = 3,795), TBX19 (dist = 29815)|chr1:168220463* (3.734080) + expression value of + expression value of SNTB2|chr16:69338598* (2.163062) + expression value of HOOK3|chr8:42883441* (3.363458) + expression value of NDUFV3|chr21:44329452* (4.443508) + expression value of FKBP11|chr12:49316769* (8.412146).

**TABLE 1:** Results of six ATIRE sites associated with the OS of patients with LUAD by multivariate Cox regression analysis.

id	Coef	HR	HR.95 L	HR.95H	*p*-Value
PGPEP1|chr19:18476416	2.005077	41.916957	6.502484	270.209250	<0.001
ANKRD36BP1(dist = 3,795),TBX19 (dist = 29815)|chr1:168220463	3.734080	427.561183	13.193038	13856.44197	0.000641
SNTB2|chr16:69338598	2.163062	100.1901446	6.618762	1516.607613	0.000890
HOOK3|chr8:42883441	3.363458	187.341956	12.414921	2827.002092	0.000157
NDUFV3|chr21:44329452	4.443508	180.007148	9.2669380	3496.578192	0.000601
FKBP11|chr12:49316769	8.412146	34422.4473	75.466409	15701090.07	0.000826

Abbreviations: Coef, coefficient; HR, hazard ratio; L,lower; H, High.

### 3.2 Verification of the six RNA editing sites for survival prediction

**FIGURE 3:**
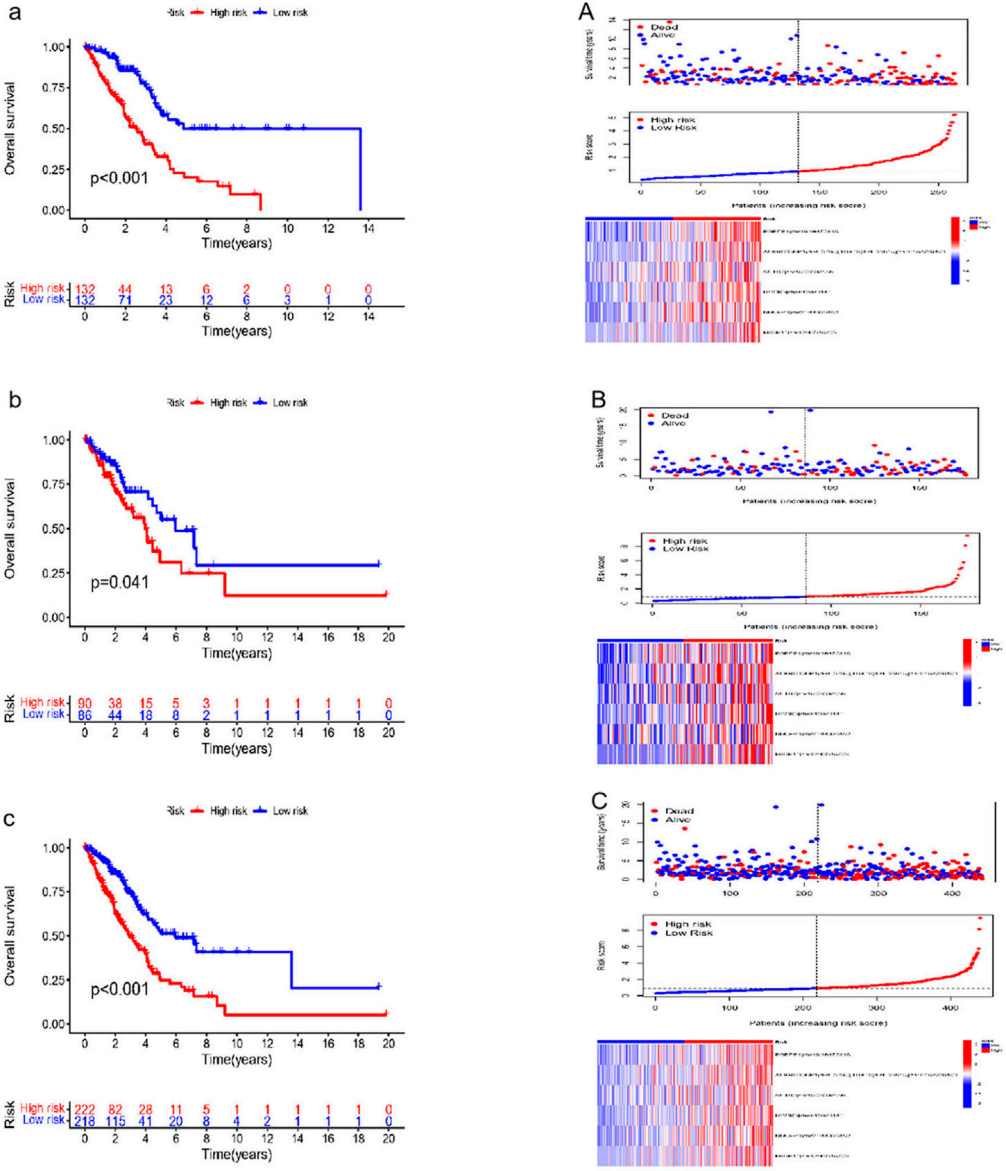
Verification of survival prediction ability and analysis of the risk score of the six ATIRE sites in LUAD. **(a,A)** Kaplan–Meier curve, survival state chart, risk curve and heatmap of expression of the RNA editing in the training set. **(b,B)** Kaplan–Meier curve, survival state chart, risk curve and heatmap of expression of the RNA editing in the validation set. **(c,C)** Kaplan–Meier curve, survival state chart, risk curve and heatmap of expression of the RNA editing in the combination set.

Six effective ATIRE sites for predicting survival were built and verified. These sites expression profiles of the TCGA LUAD cohort and the risk score calculation formula were used to determine the training set risk scores. Based on the median risk scores as the cutoff, patients were then separated into high- and low-risk groups. Patients’ mortality in the training set increased with the risk score (Figure 3a). The low-risk group had a higher survival and OS rates than that of high-risk group. A heat map of the training set also revealed the expression of six ATIRE sites (Figure 3A). The accuracy of the prediction model was also assessed through the validation and combination cohorts. Patients from the validation and combination cohorts were further separated into high-risk and low-risk groups, based on the median risk scores of the training set. Our findings indicated that patients’ mortality increased in the validation and combination groups with the risk scores (Figures 3b, c). We showed that the group with low-risk displayed a higher rate of survival, compared with the high-risk group, whereas the heat map that displayed the expression of six ATIRE sites in the validation and combination sets also demonstrated that the low-risk group’s OS was higher than high-risk group (Figure 3B, C).

### 3.3 Assessment of the prognostic value of clinical parameters

**FIGURE 4:**
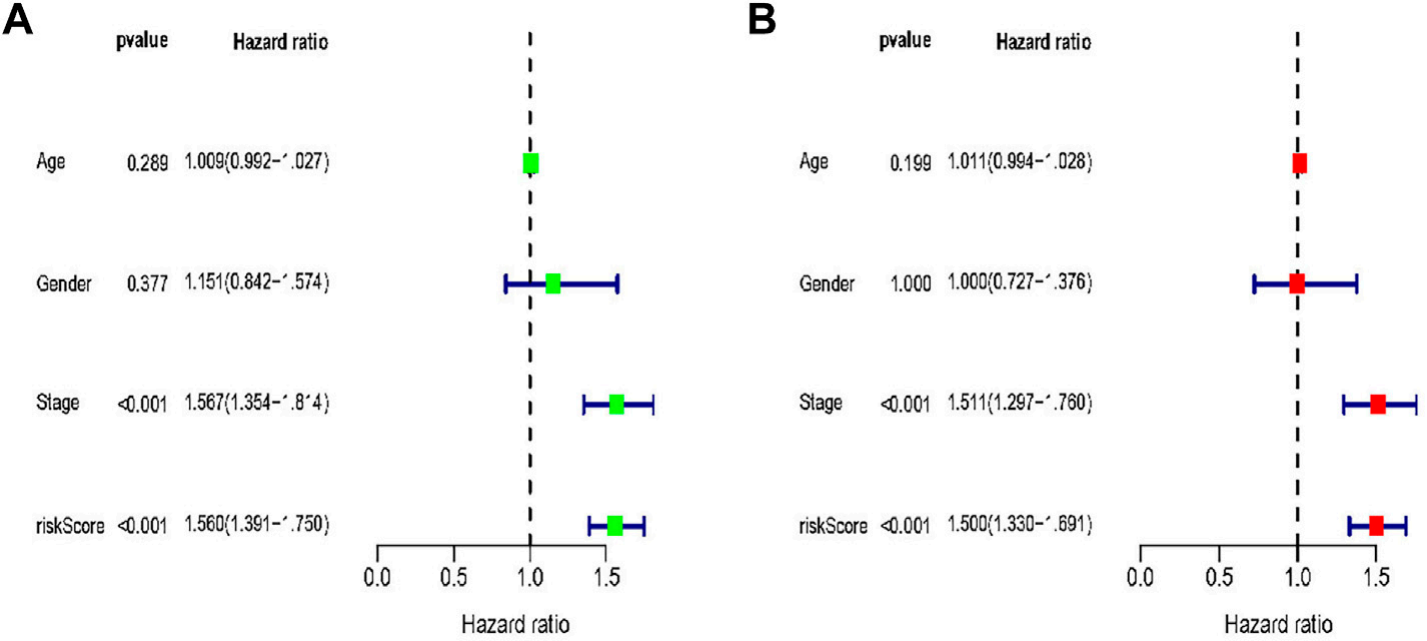
Univariate and multivariate Cox analysis of the clinical features of LUAD patients in combination sets. **(A)** Univariate Cox analysis of the clinical features of LUAD patients. **(B)** Multivariate Cox analysis of the clinical features of LUAD patients.

We used the Cox regression analysis to further evaluate the prognostic significance of various clinical traits in LUAD patients from the TCGA database. In LUAD patients, the results of univariate analysis and multivariable Cox regression analysis revealed a relationship between tumour stage and risk score and OS (Figures 4A, B).

### 3.4 Establishment and assessment of a nomogram

**FIGURE 5:**
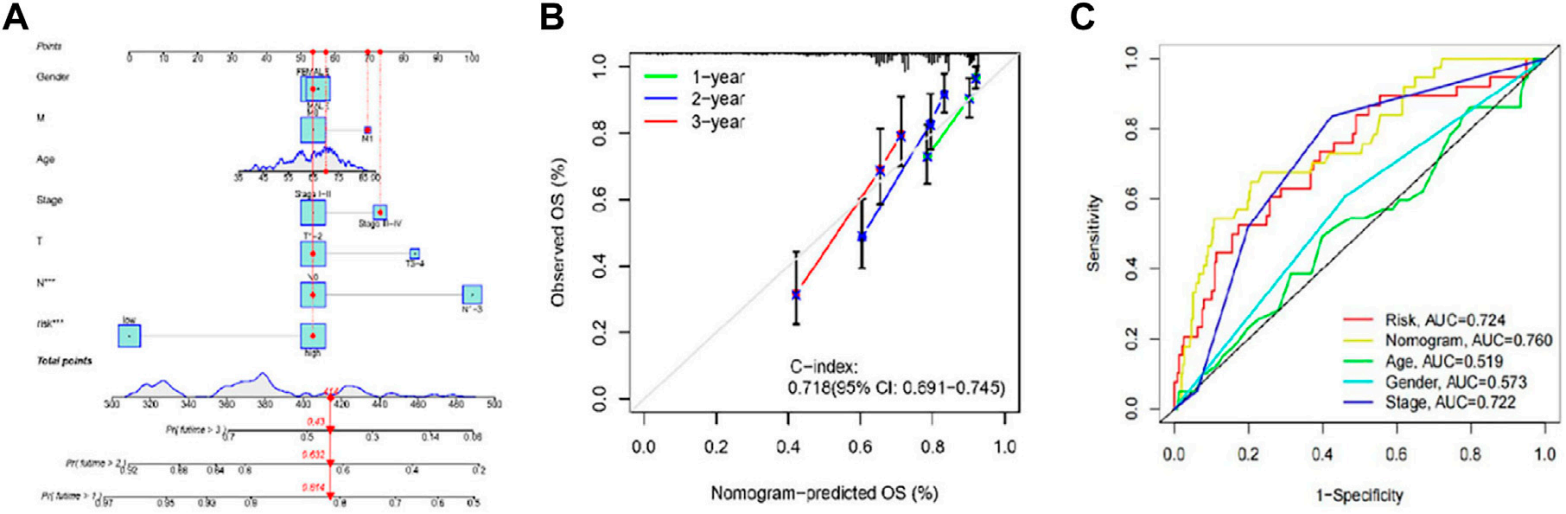
Performance of prognostic nomogram based on the RNA editing risk score and clinicopathological features. **(A)** The nomogram for predicting probabilities of 1-, 2- and 3-year OS in patients with LUAD; **(B)** Calibration curves show the agreement between the observed OS rate and nomogram-predicted OS rate at 1-, 2-, and 3-year in the training group. **(C)** The receiver operating characteristic (ROC) curve revealed that the AUC of risk score, nomogram model, age, gender, and tumor stage.

We established a nomogram with the ATIRE risk score and clinicopathological features, including risk score, age, gender, and tumor stage (Figure 5A). The C-index (0.718) and calibration plot results demonstrated the high accuracy of nomogram predictions (Figure 5B). Moreover, the ROC curve revealed that the AUC of risk score, nomogram model, age, gender, and tumor stage were 0.724, 0.760, 0.519, 0.573, and 0.772, respectively (Figure 5C).

### 3.5 Differentially expressed genes (DEGs) identification

**FIGURE 6:**
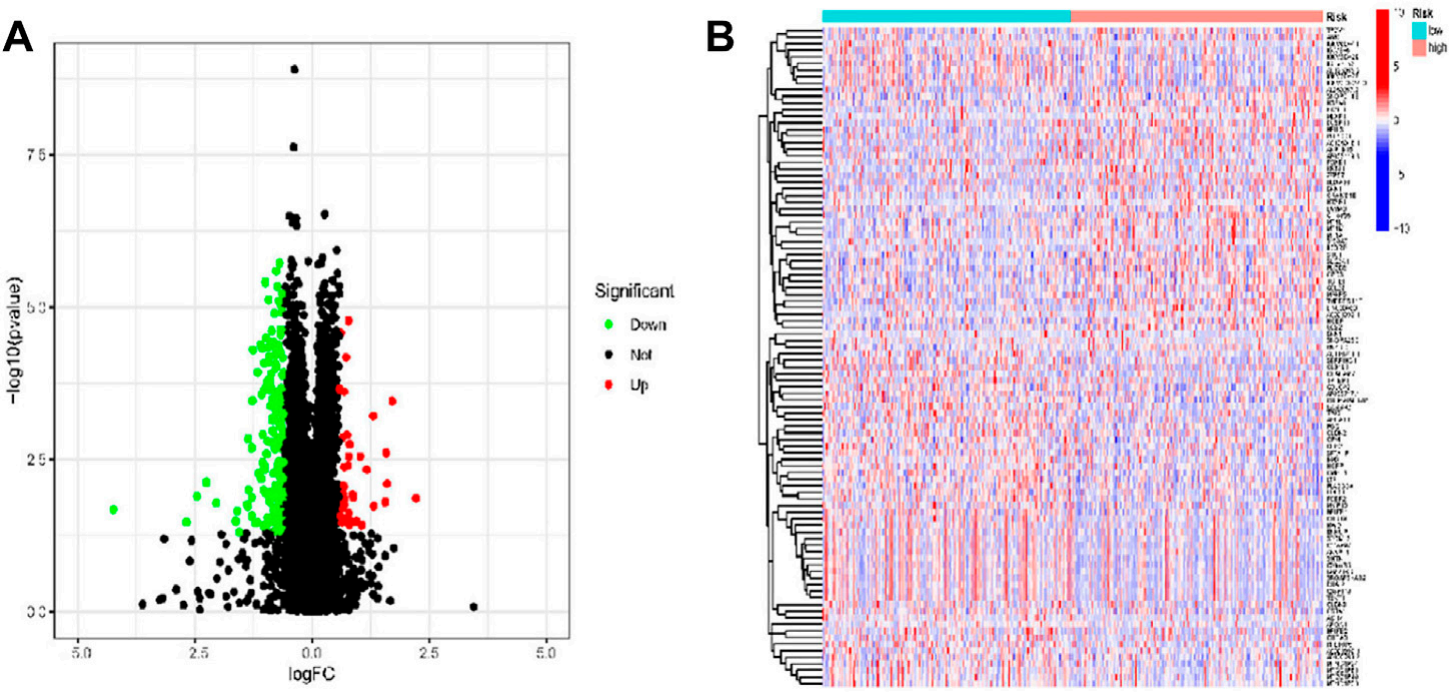
DEGs were identified from low- and high-risk groups based on TCGA data in patients with LUAD. **(A)** Volcano of the DEGs in LUAD. **(B)** Heatmap of the DEGs in LUAD.

To explore the molecular mechanism and differential functional pathway between high- and low-risk group patients, we first analyzed differential expressed genes between high-risk and low-risk groups. LUAD patients between low- and high-risk groups revealed 216 DEGs, which met the criteria of *p* < 0.05 and |log2 FC)| > 1.5. We visualized 177 genes that were upregulated and 39 genes that were downregulated, using Cytoscape. Figures 6A, B displays the clustering heatmap and volcano plot of these DEGs, between high-risk and low-risk groups.

### 3.6 Functional enrichment analysis

**FIGURE 7:**
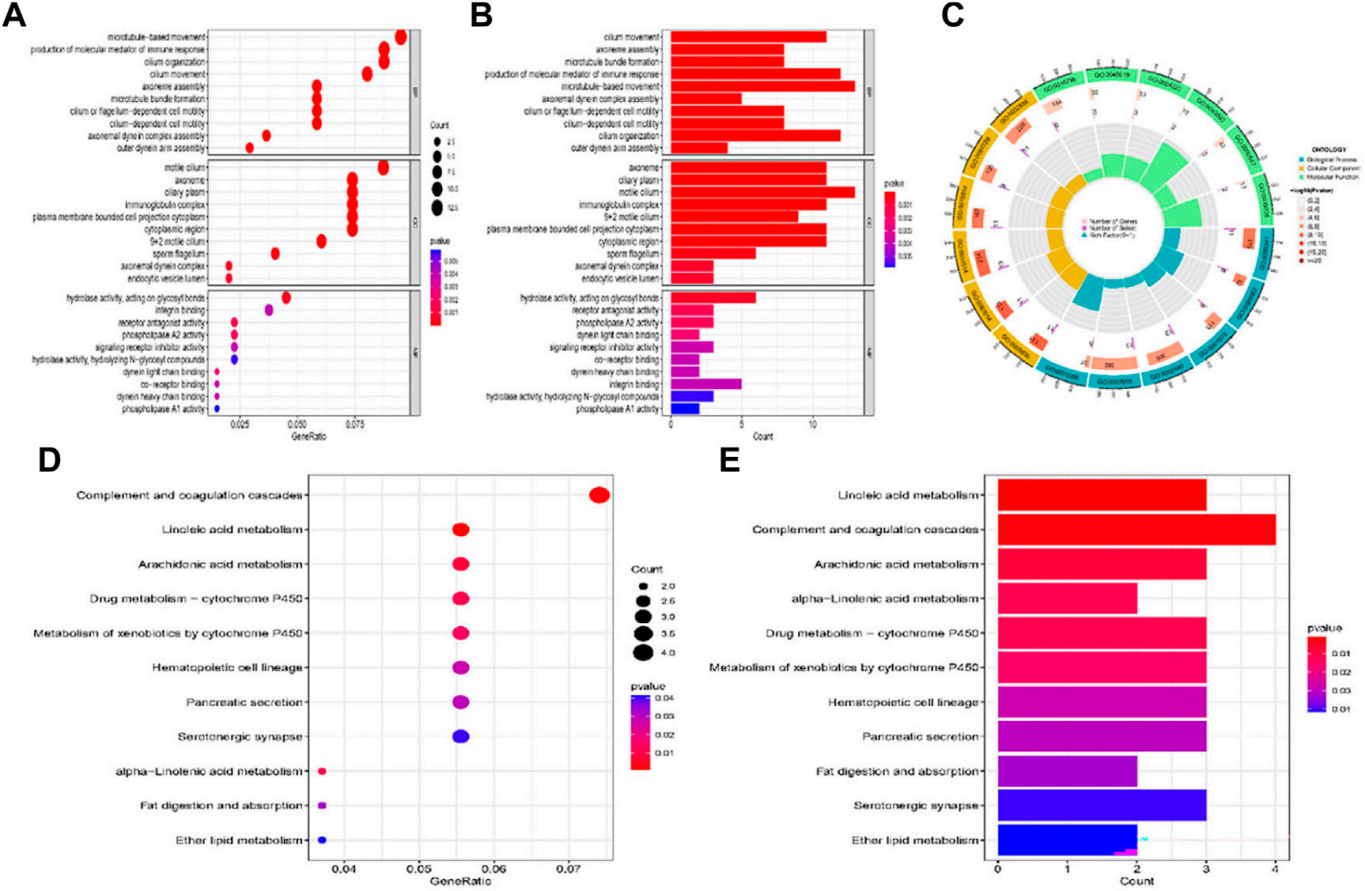
GO and KEGG pathway enrichment analysis of the DEGs between high-risk and low-risk group patients. **(A–C)** GO analysis of the DEGs between high-risk and low-risk group. **(D,E)** KEGG pathway enrichment analysis of DEGs between high-risk and low-risk group.

GO analysis suggested that DEGs were located in the cilium movement, axoneme assembly, production of molecular mediator of immune response and microtubule in certain biological processes. The cellular component analysis indicated that DEGs were all primarily enriched in axoneme and ciliary plasm, whereas the molecular function was enriched for hydrolase activity, acting on glycosyl bonds, receptor antagonist activity, and phospholipase A2 activity (Figures 7A–C). The top three KEGG pathway analysis showed that DEGs were related to complement and coagulation cascades, and linoleic acid and arachidonic acid metabolisms (Figure 7D, E).

### 3.7 Evaluation of signaling pathways

**FIGURE 8:**
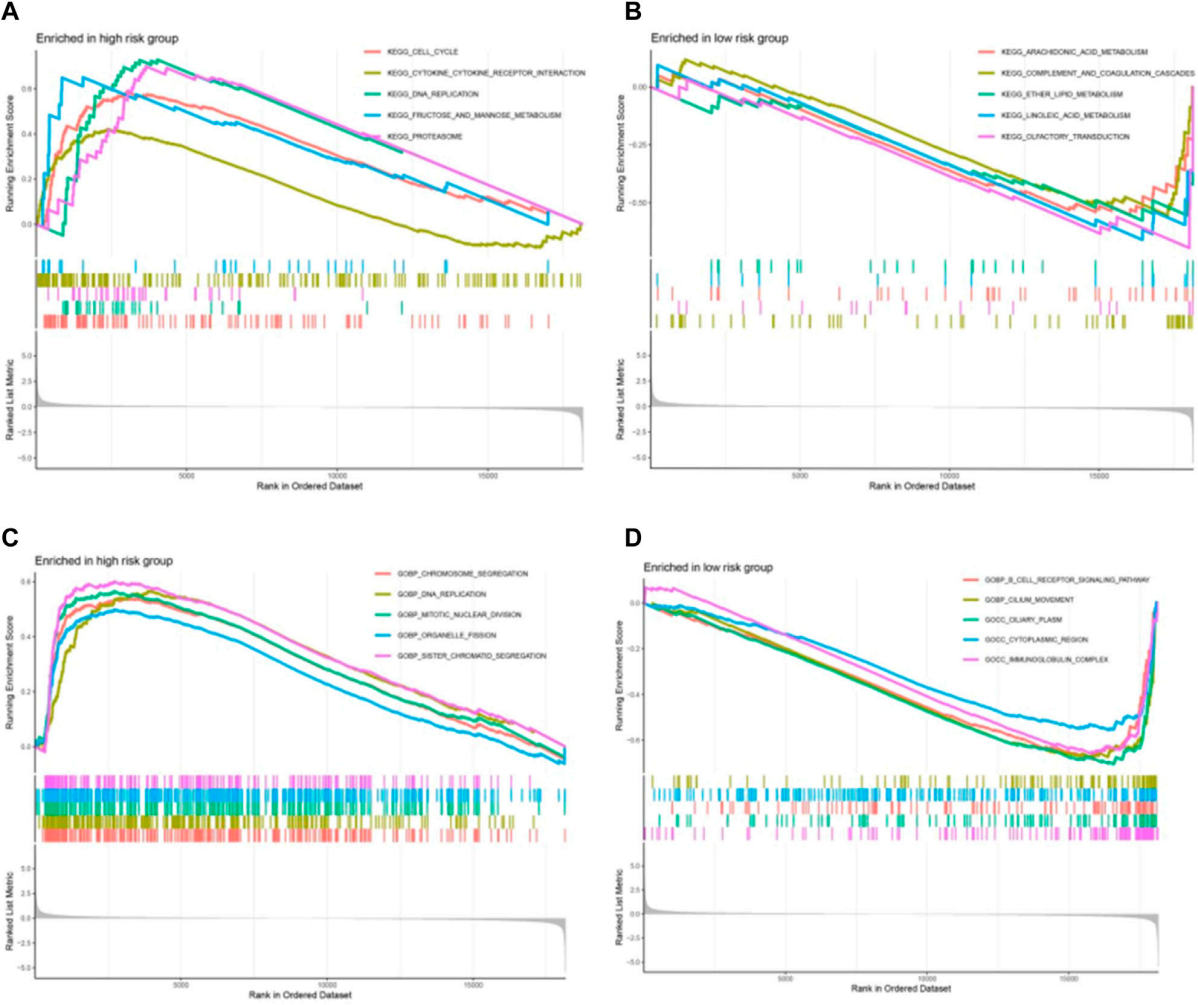
The biological function and enriched pathways between high-risk and low-risk group patients by gene set enrichment analysis (GSEA). **(A, B)** The enriched pathways between high-risk and low-risk group. **(C, D)** The biological function between high-risk and low-risk group patients.

GSEA was used to further explore the biological function and enriched pathways between high- and low-risk group patients. It showed that in the high-risk group were highly expressed mainly in cell cycle, proteasome, DNA replication, fructose and mannose metabolism pathway, and cytokine-cytokine receptor interaction (Figure 8A). In the low-risk group these genes were associated with arachidonic acid metabolism, *complement and coagulation cascades,* ether lipid metabolism and *pyrimidine metabolism* (Figure 8B). As shown in Figure 8C, the top 5 biological process in the high-risk group were chromosome segregation, DNA replication, mitotic nuclear division, organelle fission nuclear division and chromatid segregation. As shown in Figure 8D, the top 5 biological process in the low-risk group were B-cell receptor signaling pathway, cilium movement, ciliary plasm, cytoplasmic region and immunoglobulin complex.

### 3.8 Association of RNA editing risk score with ADAR gene expression

Since ATIRE is mostly mediated by ADARs, in TCGA-LUAD tumour tissues, we observed a significant correlation between the ATIRE risk score and ADAR gene expression (r = 0.24, *p* < 0.001).

### 3.9 Levels of RNA editing risk scores in tumor and normal tissue samples

**FIGURE 9:**
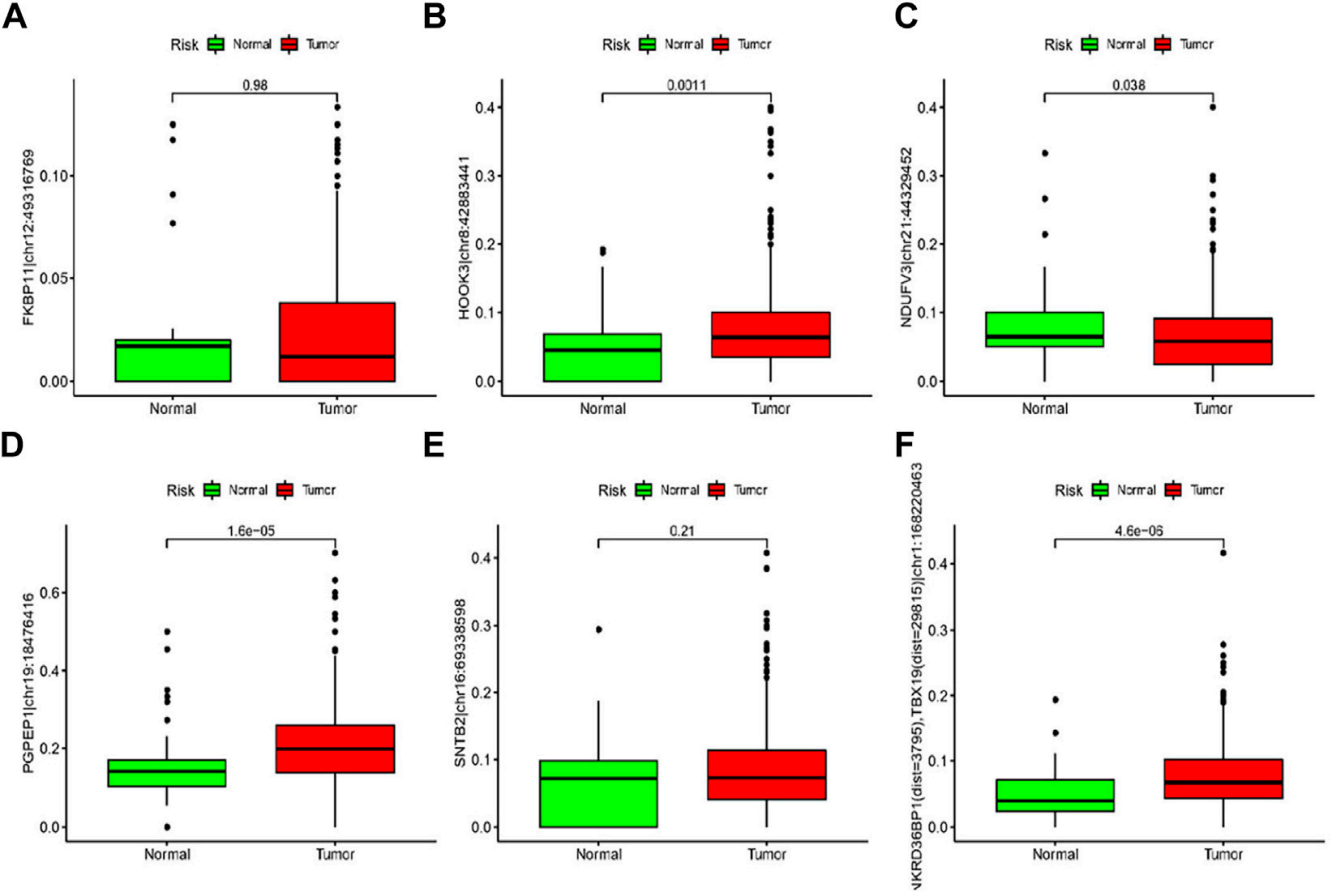
**(A–F)** The levels of six RNA editing risk scores in tumor and normal tissue samples.

As shown in Figures 9A–E, ANKRD36BP1 (dist = 3,795), TBX19 (dist = 29815) chr1:168220463A > I (*p* < 0.001), HOOK3 chr8:42883441A > I (*p* = 0.0011), PGPEP1 chr19:18476416A > I (*p* < 0.001), and NDUFV3 chr21:44329452A > I (*p* = 0.038) were significantly different between tumor and normal tissues. Tumor showed upregulation of HOOK3 chr8:42883441A > I, PGPEP1 chr19:18476416A > I, and NDUFV3 chr21:44329452A > I, as compared with normal tissue. Other ATIRE sites did not significantly differ between tumor and normal tissues (*p* > 0.05).

## 4 Discussion

As a complex illness with a dismal prognosis, LUAD is influenced by numerous genetic mutations. In this study, we systematically identified ATIRE events in LUAD and discovered that LUAD samples had a significantly higher percentage of ATIRE sites, as compared with normal tissue. Six ATIRE sites were used in our condensed model to indicate LUAD poor prognosis. The potential for clinical and therapeutic applications was indicated by the characterization of the six ATIRE sites, showing high editing levels, when the prediction model was applied.

RNA editing is closely related to the pathogenesis of many kinds of tumors as an Epigenetics mechanism. RNA editing is an important physiological process for the body to maintain normal metabolic activities and represents one of the most important mechanisms to generate the diversity and complexity of biomolecules. An essential method of genetic regulation known as RNA editing modifies RNA nucleotides, without changing the template genomic DNA (Nishikura, 2016). The significance of RNA editing events in the tumorigenesis of LUAD and the use of RNA editing in molecular subtyping were highlighted (Wang et al., 2022). Some RNA editing events might function as “driver events” for the development and spread of cancer. Diverse diseases are related to abnormal RNA editing. A-to-I RNA editing may represent an epigenetic process underlying the emergence and spread of cancer (Paz et al., 2007). During cancer initiation and progression, the level of specific gene editing is selective, as editing affects RNA levels, RNA localization, alternative splicing, translation efficiency, and protein structure and function (Levanon et al., 2004). In tumors associated with normal tissues, a striking diversity in the altered RNA-editing patterns was revealed by Han et al. through the characterization of the global A-to-I RNA editing profiles of around 17 cancer types from 6,236 patient samples (Han et al., 2015).

We found six LUAD-related ATIRE sites with potential to predict the clinical outcome in LUAD patients. The poor prognostic of LUAD patients was predicted using several parameters as suggested in the ROC curve analysis, with AUC of 0.724 for risk score, 0.760 for nomogram model, 0.519 for age, 0.573 for gender, and 0.772 for tumor stage. Outcomes demonstrated the model’s dependability in estimating clinical patients’ prognosis. This model is believed to pave the way for guiding LUAD patients with customized treatments and in the development of new biomarkers. Moreover, after duly considering the tumor stages, age, and gender, they would have independent powers for prediction. We also screened the differential ATIRE sites between LUAD and adjacent normal tissues, including editing sites in ANKRD36BP1 (dist = 3,795), TBX19 (dist = 29815) chr1:168220463A > I, HOOK3 chr8:42883441A > I, PGPEP1 chr19:18476416A > I, and NDUFV3 chr21:44329452A > I. ATIRE majorly regulates physiological and pathological processes by affecting host gene expression. However, further research is necessary to determine the exact mechanism of these editing sites. TMEM120B, HMOX2, CALCOCO2, LONP2, ZNF440, CLCC1, and CHMP3 were identified to be optimal prognostic factors for lung squamous cell carcinoma (LUSC) (Liu et al., 2022a). In multivariate Cox regression analysis, PGPEP1 chr19:18476416A > I, ANKRD36BP1 (dist = 3,795), TBX19 (dist = 29815) chr1:168220463A > I, SNTB2 chr16:69338598A > I, HOOK3 chr8:42883441A > I, NDUFV3 chr21:44329452A > I, and FKBP11 chr12:49316769A > I were prognostic ATIRE sites of LUAD patients. L-pyroglutamyl residues that sensitize the modified peptides and proteins to be degraded by the other proteases, may be removed through a hydrolytic process by the enzyme PGPEPI, whose genes have been recognized as cancer driver genes. Furthermore, SNTB2 plays a critical role in the radioresistance of cancer cells (Im et al., 2013). In prostate cancer, high levels of HOOK3 protein expression are independently associated with a poor prognosis, a poor tumour phenotype, and an early PSA recurrence (Melling et al., 2015). TBX19 gene encodes a transcription factor characterized by a highly conserved DNA-binding motif. Recent research has shown that TBX19 gene, which is increased in colon adenomas, was identified as one of the genes triggered by KRAS mutations (Ando et al., 2017). When treating patients with clear cell renal cell carcinoma, NDUFV3 was found to be an independent predictor of overall survival (Jia et al., 2018), whereas FKBP11 has the potential to be an early marker for hepatocellular carcinoma (Lin et al., 2013). In non-coding regions, most ATIRE sites are in introns and repetitive Alu elements embedded in 3′untranslated regions (3′UTRs) (Qi et al., 2017). The biological significance of editing within non-coding regions of RNA is still poorly understood. In prostate cancer, ATIRE alters the interaction of androgen receptor with androgens or anti-androgenic ligands (Martinez et al., 2008). In liver cancer, editing of the antizyme inhibitor AZIN1 induces its cytoplasmic-to-nuclear translocation to increase tumor aggressiveness (Chen et al., 2013), whereas in colorectal cancer, ATIRE impacts Ras homolog family member Q (RHOQ) to promote invasion (Han et al., 2014). Moreover, ADAR1 and global RNA editomes were elevated in glioblastoma patients. ADAR1 inactivation or blocking of the upstream JAK/STAT pathway through TYK2 inhibition impaired GSC self-renewal and stemness (Jiang et al., 2022).

ADAR family genes were originally identified as the major regulators of RNA editing events. Accumulating evidence now supports the role of Adar-mediated ATIRE in cancer development and progression (Chan et al., 2016). The link between RNA editing and cancer is more complex because ATIRE enzymes are both tumor suppressors and oncogenes. Furthermore, ADAR1 may play a role in protecting the body from cardiac damage caused by interferon activation, which is related to chronic inflammation, automatic immune diseases, and cancer. Numerous reports have shown that RNA editing is related to human diseases. In addition to causing coding alterations that support tumors, ADAR works to protect them by suppressing ISG-triggered immune responses and promoting tumor cell survival (Nemlich et al., 2013; Herbert, 2019). As a result of CDK13 editing, the protein is more abundant in nucleoli, and this phenomenon may account for at least some of the worldwide change in splicing brought on by ADAR1 deregulation (Ramírez-Moya et al., 2021). Multiple malignancies, including hepatocellular carcinoma, chronic myelogenous leukemia, glioblastoma, and melanoma have aberrant ADAR activity and editing dysregulation (Wang et al., 2017b). The imbalance of ADAR expression or activity may lead to a variety of diseases, such as cancer, Aicardi–Goutières syndrome, and amyotrophic lateral sclerosis (Galeano et al., 2012). Recent research demonstrated that ADAR1 plays a significant role in the homeostatic regulation of the gastrointestinal system, skin, and bone (Yu et al., 2013). ADARs enzymes catalyze the conversion of A-to-I in double-stranded RNA during RNA editing in higher eukaryotes. Initially, it was believed that ADAR enzymes only functioned in certain genes’ coding regions (Chen et al., 2013). ADAR2 is associated with a variety of tumors, inflammation, lupus erythematosus, amyotrophic lateral sclerosis, Alzheimer’s disease, and other diseases (Mannion et al., 2015), whereas ADAR1 expression levels and site-specific editing levels may function as prognostic biomarkers for certain cancer types, because they have a strong correlation with cancer patients’ OS (Han et al., 2015; Wang et al., 2022). Cancer cells with altered RNA editing have a selective advantage in tumor growth and apoptosis resistance. Cancer developing is facilitated by RNA editing through dynamically recoding carcinogenic genes (Baysal et al., 2017). Reduced ADARs expression is not limited to editing but extends to other activities such as inhibition of dsRNA-activated protein kinase PKR kinase activity and inhibition of eIF2a-dependent ADAR phosphorylation by mechanisms dependent on dsRNA editing (Nie et al., 2007).

We further investigated the bioinformatics of aberrantly expressed RNA editing and differences in molecular mechanisms and functional pathways between high-risk and low-risk patients. According to the results of the GO biological process keywords, DEGs between high- and low-risk group mostly focus on cilium movement, axoneme assembly, and microtubules. Molecular functions were mainly enriched for hydrolase activity, acting on glycosyl bonds, receptor antagonist activity, and phospholipase A2 activity. The top three KEGG pathway analysis showed that DEGs were related to the complement and coagulation cascades, and linoleic acid and arachidonic acid metabolisms.

GSEA was used to further explore the biological function and enriched pathways between high- and low-risk group patients. It showed that in the high-risk group were highly expressed mainly in cell cycle, proteasome, DNA replication, fructose and mannose metabolism pathway, and cytokine-cytokine receptor interaction, which are closely related to the proliferation and growth of cancer cells, which may be one of the reasons for the poor prognosis of high-risk groups. In high-risk group tend to affect targets involved in cancer-related signaling pathways and processes, such as cell cycle and DNA replication. Deregulation of the proteasome pathway plays important roles in the pathogenesis of lung cancer (Kakumu et al., 2017). Cytokine-cytokine receptor interaction is an important immune system signaling pathway as it regulates cytokine interactions and thus cancer progression (Dranoff, 2004). RNA editing contributes to peptide diversity and editing-derived epitopes may elicit immune responses in cancer cells (Zhang et al., 2018). As shown by the GSEA results, low-risk group enriched many pathways related to arachidonic acid metabolism, complement and coagulation cascades, ether lipid metabolism and pyrimidine metabolism, indicating that metabolism was strongly associated with LUAD patients with a low-risk score. The arachidonic acid pathway is a metabolic process that plays a key role in carcinogenesis, and its enzymes are emerging as novel targets for cancer prevention and therapy (Yarla et al., 2016). Simultaneously weakening the complement system and the coagulation cascade seems to be a prudent treatment for cancer patients (Liu et al., 2022b). The pyrimidine pathway contributes to cancer mechanisms (Weber, 1983).

The analysis of editing sites in LUAD is expected to provide new markers for the diagnosis and prognosis of cancer. We demonstrated editing sites associated with LUAD by contrasting LUAD cancer and normal samples. Thus, as a complimentary event to DNA mutation in LUAD risk genes, we showed that ATIRE may be a risk factor for LUAD. The RNA editing-based model may be used as a unique method for predicting LUAD survival.

## Data Availability

The original contributions presented in the study are included in the article/Supplementary Material, further inquiries can be directed to the corresponding author.

## References

[B1] Ando J. Saito M. Imai J. I. Ito E. Yanagisawa Y. Honma R. (2017). TBX19 is overexpressed in colorectal cancer and associated with lymph node metastasis. Fukushima J. Med. Sci. 63 (3), 141–151. 10.5387/fms.2017-08 29199261 PMC5792498

[B2] Baysal B. E. Sharma S. Hashemikhabir S. Janga S. C. (2017). RNA editing in pathogenesis of cancer. Cancer Res. 77 (14), 3733–3739. 10.1158/0008-5472.CAN-17-0520 28667076

[B3] Bazak L. Haviv A. Barak M. Jacob-Hirsch J. Deng P. Zhang R. (2014a). A-to-I RNA editing occurs at over a hundred million genomic sites, located in a majority of human genes. Genome Res. 24, 365–376. 10.1101/gr.164749.113 24347612 PMC3941102

[B4] Chan T. H. Qamra A. Tan K. T. Guo J. Yang H. Qi L. (2016). ADAR-mediated RNA editing predicts progression and prognosis of gastric cancer. Gastroenterology 151 (4), 637–650. 10.1053/j.gastro.2016.06.043 27373511 PMC8286172

[B5] Chen L. Li Y. Lin C. H. Chan T. H. M. Chow R. K. K. Song Y. (2013). Recoding RNA editing of AZIN1 predisposes to hepatocellular carcinoma. Nat. Med. 19 (2), 209–216. 10.1038/nm.3043 23291631 PMC3783260

[B6] Chigaev M. Yu H. Samuels D. C. Sheng Q. Oyebamiji O. Ness S. (2019). Genomic positional dissection of RNA editomes in tumor and normal samples. Front. Genet. 10, 211. 10.3389/fgene.2019.00211 30949194 PMC6435843

[B7] Dranoff G. (2004). Cytokines in cancer pathogenesis and cancer therapy. Nat. Rev. Cancer 4 (1), 11–22. 10.1038/nrc1252 14708024

[B8] Fritzell K. Xu L. D. Otrocka M. Andréasson C. Öhman M. (2019). Sensitive ADAR editing reporter in cancer cells enables high-throughput screening of small molecule libraries. Nucleic Acids Res. 47 (4), e22. 10.1093/nar/gky1228 30590609 PMC6393238

[B9] Galeano F. Tomaselli S. Locatelli F. Gallo A. (2012). A-to-I RNA editing: The "ADAR" side of human cancer. Semin. Cell Dev. Biol. 23, 244–250. 10.1016/j.semcdb.2011.09.003 21930228

[B10] Han L. Diao L. Yu S. Xu X. Li J. Zhang R. (2015). The genomic landscape and clinical relevance of A-to-I RNA editing in human cancers. Cancer Cell 28 (4), 515–528. 10.1016/j.ccell.2015.08.013 26439496 PMC4605878

[B11] Han S. W. Kim H. P. Shin J. Y. Jeong E. G. Lee W. C. Kim K. Y. (2014). RNA editing in RHOQ promotes invasion potential in colorectal cancer. J. Exp. Med. 211 (4), 613–621. 10.1084/jem.20132209 24663214 PMC3978269

[B12] Herbert A. (2019). ADAR and immune silencing in cancer. Trends Cancer 5 (5), 272–282. 10.1016/j.trecan.2019.03.004 31174840

[B13] Hu X. Chen J. Shi X. Feng F. Lau K. W. Chen Y. (2017). RNA editing of AZIN1 induces the malignant progression of non-small-cell lung cancers. Tumour Biol. 39, 8. 10.1177/1010428317700001 28849733

[B14] Huntley M. A. Lou M. Goldstein L. D. Lawrence M. Dijkgraaf G. J. P. Kaminker J. S. (2016). Complex regulation of ADAR-mediated RNA-editing across tissues. BMC genomics 17 (15), 61. 10.1186/s12864-015-2291-9 26768488 PMC4714477

[B15] Im C-N. Kim B. M. Moon E. Y. Hong D. W. Park J. W. Hong S. H. (2013). Characterization of H460R, a radioresistant human lung cancer cell line, and involvement of Syntrophin Beta 2 (SNTB2) in radioresistance. Genomics & Inf. 11, 245–253. 10.5808/GI.2013.11.4.245 PMC3897853 24465237

[B16] Jia Z. Wan F. Zhu Y. Shi G. Zhang H. Dai B. (2018). Forkhead-box series expression network is associated with outcome of clear-cell renal cell carcinoma. Oncol. Lett. 15 (6), 8669–8680. 10.3892/ol.2018.8405 29805604 PMC5950509

[B17] Jiang L. Hao Y. Shao C. Wu Q. Prager B. C. Gimple R. C. (2022). ADAR1-mediated RNA editing links ganglioside catabolism to glioblastoma stem cell maintenance. J. Clin. Invest. 132, e143397. 10.1172/JCI143397 35133980 PMC8920333

[B18] Kakumu T. Sato M. Goto D. Kato T. Yogo N. Hase T. (2017). Identification of proteasomal catalytic subunit PSMA6 as a therapeutic target for lung cancer. Cancer Sci. 108, 732–743. 10.1111/cas.13185 28165654 PMC5406588

[B19] Kung C-P. Maggi L. B. Weber J. D. (2018). The role of RNA editing in cancer development and metabolic disorders. Front. Endocrinol. 9, 762. 10.3389/fendo.2018.00762 PMC6305585 30619092

[B20] Levanon E. Y. Eisenberg E. Yelin R. Nemzer S. Hallegger M. Shemesh R. (2004). Systematic identification of abundant A-to-I editing sites in the human transcriptome.” Nat. Biotechnol. 22, 1001–1005. 10.1038/nbt996 15258596

[B21] Lin I-Y. Yen C. H. Liao Y. J. Lin S. E. Ma H. P. Chan Y. J. (2013). Identification of FKBP11 as a biomarker for hepatocellular carcinoma. Anticancer Res. 33 (6), 2763–2769. 23749938

[B22] Liu L. Liu J. Deng X. Tu L. Zhao Z. Xie C. (2022). A nomogram based on A-to-I RNA editing predicting overall survival of patients with lung squamous carcinoma. BMC cancer 22, 715. 10.1186/s12885-022-09773-0 35768804 PMC9241197

[B23] Liu X. Wang Y. Bauer A. T. Kirschfink M. Ding P. Gebhardt C. (2022). Neutrophils activated by membrane attack complexes increase the permeability of melanoma blood vessels. Proc. Natl. Acad. Sci. U. S. A. 119, 33. 10.1073/pnas.2122716119 PMC9388087 35960843

[B24] Mannion N. Arieti F. Gallo A. Keegan L. P. O'Connell M. A. (2015). New insights into the biological role of mammalian ADARs; the RNA editing proteins. Biomolecules 5, 2338–2362. 10.3390/biom5042338 26437436 PMC4693238

[B25] Martinez H. D. Jasavala R. J. Hinkson I. Fitzgerald L. D. Trimmer J. S. Kung H. J. (2008). RNA editing of androgen receptor gene transcripts in prostate cancer cells. J. Biol. Chem. 283, 29938–29949. 10.1074/jbc.M800534200 18708348 PMC2662061

[B26] Melling N. Harutyunyan L. Hube-Magg C. Kluth M. Simon R. Lebok P. (2015). High-level HOOK3 expression is an independent predictor of poor prognosis associated with genomic instability in prostate cancer. PLoS One 10, e0134614. 10.1371/journal.pone.0134614 26230842 PMC4521853

[B27] Nebbioso A. Tambaro F. P. Dell'Aversana C. Altucci L. (2018). Cancer epigenetics: Moving forward. PLoS Genet. 14, e1007362. 10.1371/journal.pgen.1007362 29879107 PMC5991666

[B28] Nemlich Y. Greenberg E. Ortenberg R. Besser M. J. Barshack I. Jacob-Hirsch J. (2013). MicroRNA-mediated loss of ADAR1 in metastatic melanoma promotes tumor growth. J. Clin. Invest. 123 (6), 2703–2718. 10.1172/JCI62980 23728176 PMC3668823

[B29] Nie Y. Hammond G. L. Yang J. H. (2007). Double-stranded RNA deaminase ADAR1 increases host susceptibility to virus infection. J. virology 81 (2), 917–923. 10.1128/JVI.01527-06 17079286 PMC1797455

[B30] Nishikura K. (2016). A-to-I editing of coding and non-coding RNAs by ADARs. Nat. Rev. Mol. Cell Biol. 17, 83–96. 10.1038/nrm.2015.4 26648264 PMC4824625

[B31] Paz N. Levanon E. Y. Amariglio N. Heimberger A. B. Ram Z. Constantini S. (2007). Altered adenosine-to-inosine RNA editing in human cancer. Genome Res. 17, 1586–1595. 10.1101/gr.6493107 17908822 PMC2045141

[B32] Paz-Yaacov N. Bazak L. Buchumenski I. Porath H. T. Danan-Gotthold M. Knisbacher B. A. (2015). Elevated RNA editing activity is a major contributor to transcriptomic diversity in tumors. Cell Rep. 13, 267–276. 10.1016/j.celrep.2015.08.080 26440895

[B33] Peng X. Xu X. Wang Y. Hawke D. H. Yu S. Han L. (2018). A-to-I RNA editing contributes to proteomic diversity in cancer. Cancer Cell 33, 5. 10.1016/j.ccell.2018.03.026 29706454 PMC5953833

[B34] Qi L. Song Y. Chan T. H. M. Yang H. Lin C. H. Tay D. J. T. (2017). An RNA editing/dsRNA binding-independent gene regulatory mechanism of ADARs and its clinical implication in cancer. Nucleic Acids Res. 45 (18), 10436–10451. 10.1093/nar/gkx667 28985428 PMC5737565

[B35] Qin Y-R. Qiao J. J. Chan T. H. M. Zhu Y. H. Li F. F. Liu H. (2014). Adenosine-to-inosine RNA editing mediated by ADARs in esophageal squamous cell carcinoma. Cancer Res. 74 (3), 840–851. 10.1158/0008-5472.CAN-13-2545 24302582

[B36] Ramírez-Moya J. Miliotis C. Baker A. R. Gregory R. I. Slack F. J. Santisteban P. (2021). An ADAR1-dependent RNA editing event in the cyclin-dependent kinase CDK13 promotes thyroid cancer hallmarks. Mol. Cancer 20(1), 115. 10.1186/s12943-021-01401-y 34496885 PMC8424981

[B37] Sharma S. Patnaik S. K. Taggart R. T. Kannisto E. D. Enriquez S. M. Gollnick P. (2015). APOBEC3A cytidine deaminase induces RNA editing in monocytes and macrophages. Nat. Commun. 6, 6881. 10.1038/ncomms7881 25898173 PMC4411297

[B38] Su A. A. H. Randau L. (2011). A-to-I and C-to-U editing within transfer RNAs. Biochem. Biokhimiia 76 (8), 932–937. 10.1134/S0006297911080098 22022967

[B39] Wang C. Huang M. Chen C. Li Y. Qin N. Ma Z. (2022). Identification of A-to-I RNA editing profiles and their clinical relevance in lung adenocarcinoma. Sci. China Life Sci. 65 (1), 19–32. 10.1007/s11427-020-1928-0 34050895

[B40] Wang C. Zou J. Ma X. Wang E. Peng G. (2017). Mechanisms and implications of ADAR-mediated RNA editing in cancer. Cancer Lett. 411, 27–34. 10.1016/j.canlet.2017.09.036 28974449

[B41] Wang Y. Xu X. Yu S. Jeong K. J. Zhou Z. Han L. (2017). Systematic characterization of A-to-I RNA editing hotspots in microRNAs across human cancers. Genome Res. 27(7), 1112–1125. 10.1101/gr.219741.116 28411194 PMC5495064

[B42] Weber G. (1983). Enzymes of purine metabolism in cancer. Clin. Biochem. 16 (1), 57–63. 10.1016/s0009-9120(83)94432-6 6861338

[B43] Xu X. Wang Y. Liang H. (2018). The role of A-to-I RNA editing in cancer development. Curr. Opin. Genet. Dev. 48, 51–56. 10.1016/j.gde.2017.10.009 29127844 PMC5869101

[B44] Yarla N. S. Sethi G. Reddanna P. Kalle A. M. Dhananjaya B. L. Dhananjaya B. L. (2016). Targeting arachidonic acid pathway by natural products for cancer prevention and therapy.” Seminars cancer Biol. 40-41, 48–81. 10.1016/j.semcancer.2016.02.001 26853158

[B45] Yu S. Sharma R. Nie D. Jiao H. Im H. J. Lai Y. (2013). ADAR1 ablation decreases bone mass by impairing osteoblast function in mice. Gene 513, 101–110. 10.1016/j.gene.2012.10.068 23123729 PMC3514579

[B46] Zhang M. Fritsche J. Roszik J. Williams L. J. Peng X. Chiu Y. (2018). RNA editing derived epitopes function as cancer antigens to elicit immune responses.” Nat. Commun. 9, 3919. 10.1038/s41467-018-06405-9 30254248 PMC6156571

